# Lysosome-Targeted Single Fluorescence Probe for Two-Channel Imaging Intracellular SO_2_ and Biothiols

**DOI:** 10.3390/molecules24030618

**Published:** 2019-02-11

**Authors:** Yue Wang, Li Liu, Xian-Li Zhou, Ming-Yu Wu

**Affiliations:** School of Life Science and Engineering, Southwest Jiaotong University, Chengdu 610031, China; chinakywy2009@163.com (Y.W.); 18782172684@163.com (L.L.); xxbiochem@163.com (X.-L.Z.)

**Keywords:** single fluorescent probe, lysosome-targeted, SO_2_, biothiols, fluorescence imaging

## Abstract

As the members of reactive sulfur species, SO_2_ and biothiols play a significant role in physiological and pathological processes and directly influence numerous diseases. Furthermore, SO_2_ and biothiols can provide a reductive environment for lysosomes to carry out their optimal functionality. To this end, the development of single fluorescent probes for imaging SO_2_ and biothiols from different emission channels is highly desirable for understanding their physiological nature. Here, a lysosome-targeted fluorescent probe (**BPO-DNSP**) with a dual reaction site for SO_2_ and biothiols was presented. **BPO-DNSP** can sensitively and selectively respond to SO_2_ in the green channel with a large Stokes shift over 105 nm, and to biothiols in the near-infrared emission channel with a large Stokes shift over 109 nm. The emission shift for the two channels was as high as 170 nm. Colocalization experiments verified that **BPO-DNSP** can selectively enrich lysosomes. Notably, **BPO-DNSP** can not only be used to image intracellular SO_2_ and biothiols from two different channels, but also to monitor the conversion of biothiols to SO_2_ without adding exogenous enzymes in living HeLa cells.

## 1. Introduction

Reactive sulfur species (RSS), such as SO_2_ and biothiols, play a critical role in physiological and pathological processes [1,2,3,4]. Biothiols, including cysteine (Cys), homocysteine (Hcy), and glutathione (GSH), can not only serve as unique units in intracellular signal transduction but can also maintain redox homeostasis in the biological environment [5,6,7,8]. The intracellular biothiol concentration can be as high as 1–10 mM [2,9]. Abnormal levels of biothiols are critical for many diseases. For example, the deficiency of biothiols can lead to lethargy, liver damage, psoriasis, and slow growth in children, whereas high levels of biothiols are considered to be associated with Alzheimer’s disease, cardiovascular disease, osteoporosis, and cancers [10,11,12]. SO_2_ is a potential signal transmitter generated endogenously via biosynthetic pathways in vivo [3,13,14]. It has been reported that SO_2_ is directly related to symptoms of neurological disorders, cardiovascular diseases, and lung cancer [15]. Furthermore, biothiols are essential molecules which are the source of other RSS, and SO_2_ can be generated endogenously from biothiols [13]. The metabolism of biothiols with dioxygenase as a catalyst can generate cysteinesulfinate which further converts to β-sulfinylpyruvate and then decomposes to SO_2_ by aspartate aminotransferase [16]. Nevertheless, it would be still highly valuable to better understand their relationship and cellular cross-talk. Thus, to investigate the pathological mechanisms of disease, it is of great importance to monitor both SO_2_ and biothiols with high sensitivity and selectivity in living cells.

Fluorescence technology is a powerful tool applied in the biological field due to its simple operation and high sensitivity as well as excellent spatial and temporal resolution [17,18,19,20]. Till now, numerous fluorescent probes have been developed for detecting biothiols or SO_2_ independently [16,19,21,22,23]. To better understand their physiological nature and cellular cross-talk, a single fluorescent probe with dual functions could provide more detailed information about the microenvironment and physical condition of the cells [24]. However, there are still few fluorescent probes presented for imaging SO_2_ and biothiols with distinct responses from different emission channels [25,26,27,28].

RSS widely exist in cytoplasm and different organelles, such as mitochondria, lysosomes, endoplasmic reticulum, and so on. Being an important organelle found in nearly all eukaryotic cells, lysosomes play an important role in intracellular digestion and cellular apoptosis—a function that requires a highly reductive environment provided by RSS for optimal functionality [29,30,31]. RSS, especially biothiols, can stabilize the lysosomal membrane, stimulate albumin degradation in liver lysosomes, and facilitate intralysosomal hydrolase [32,33]. Nevertheless, the introduction of oxidative stress or metabolic dysfunction affects biothiol and SO_2_ concentrations in the lysosome, and this could be a possible indicator of disease states [34]. Therefore, fluorescent probes that respond to SO_2_ and biothiols within the lysosomes could provide deep insights into their specific roles in the physiological and pathological process [35,36,37]. On this account, the development of lysosome-targeted, single, fluorescent probes for separate detection of SO_2_ and biothiols with high sensitivity and selectivity is highly desirable.

## 2. Results and Discussion

### 2.1. Design Strategy and Synthesis of Probes

**Scheme 1:**
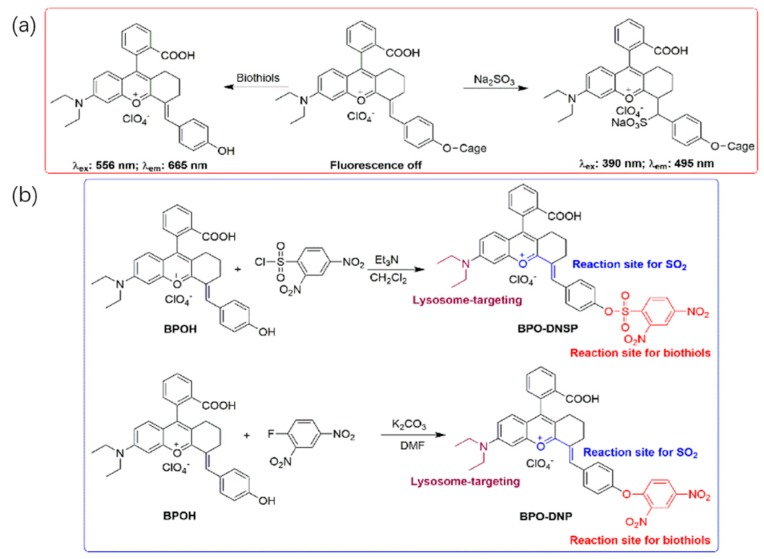
Design strategy and synthesis routes of a lysosome-targeted fluorescent probe (**BPO-DNSP**) and **BPO-DNP**. (**a**) The reaction mechanisms of the fluorescent probe for SO_2_ and biothiols; (**b**) Synthesis routes of **BPO-DNSP** and **BPO-DNP**.

Frequently, strong, electron-withdrawing groups such as 2,4-dinitrofluorobenzene or 2,4-dinitrobenzenesulfonyl chloride were often used to cage the phenolic hydroxyl of chromophore and could be cleaved through a nucleophilic substitution reaction by thiols to release the fluorescence [38,39,40,41]. Meanwhile, the carbon–carbon double bond linked with strong electron-withdrawing groups was frequently used as a recognition site for SO_2_ [42,43,44,45,46,47]. Bearing these facts in mind, chromenylium with a phenolic hydroxyl group (**BPOH**) was a rational design caged with 2,4-dinitrobenzenesulfonyl chloride and 2,4-dinitrofluorobenzene to synthesize **BPO-DNSP** and **BPO-DNP**, respectively, as depicted in Scheme 1. Moreover, the caging group can regulate the charge distribution of the carbon–carbon double bond, which can improve the sensitivity and selectivity for SO_2_ detection. At the same time, the necessary molecular decoration for lysosome targeting was incorporated via the *N*,*N*-diethyl group according to the literature [33,48,49,50]. Thus, **BPO-DNSP** and **BPO-DNP** were synthesized (Scheme 1b) and anticipated to separate the response of SO_2_ and biothiols in lysosomes from distinct fluorescent signals with high sensitivity and selectivity. The structures of **BPO-DNSP** and **BPO-DNP** were characterized by ^1^H NMR, ^13^C NMR, and HRMS.

### 2.2. Separate Response of Probes to SO_2_ and Biothiols in Different Channels

**Figure 1:**
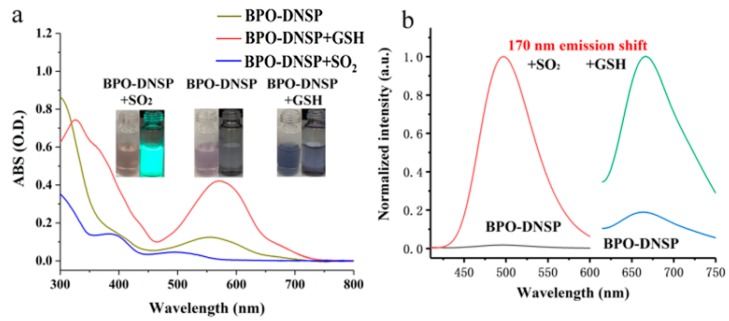
(**a**) UV-vis absorption and (**b**) normalized fluorescence spectra of **BPO-DNSP** (10 μM) and its interaction with 500 μM SO_2_ or glutathione (GSH). Excitation wavelength for **BPO-DNSP** with SO_2_ (λ_ex_) = 390 nm. Slit: 2.5 nm/5 nm. Excitation wavelength for **BPO-DNSP** with GSH (λ_ex_) = 556 nm. Slit: 10 nm/10 nm. Inset: corresponding color and fluorescence changes under visible light and a 365 nm UV lamp.

UV-vis absorption and fluorescence spectra were explored to evaluate the optical response of **BPO-DNSP** or **BPO-DNP** to SO_2_ and biothiols in different channels. **BPO-DNSP** showed a maximum absorption peak at approximately 556 nm in a phosphate-buffered saline (PBS) and dimethylsulfoxide (DMSO) mixture solution (8:2, *v*/*v*). After the addition of 500 μM SO_2_, the 556 nm absorption peaks shifted to 500 nm (Figure 1a), which suggested that SO_2_ indeed reacted with **BPO-DNSP** and broke the conjugated system. As a comparison, after adding 500 μM GSH, the absorption peak shifted from 556 nm to 566 nm and was accompanied by an increased absorption intensity (Figure 1a).

Thereafter, the fluorescence responses of **BPO-DNSP** to SO_2_ and biothiols in the same system were investigated. As depicted in Figure 1b, **BPO-DNSP** showed almost no fluorescence at 495 nm and 665 nm with the excitation at 390 nm and 556 nm, respectively. The fluorescence quantum yields were 0.003 and 0.0026 using fluorescein as a reference (Φ_s_ = 0.85 in 0.1M NaOH aqueous solution). However, after treatment with SO_2_, the emission intensity centred at 495 nm increased significantly (Figure 1b), with green fluorescence increasing observably under 365 nm excitation UV light (Figure 1a) and the quantum yield increasing up to 0.664. On the other hand, after treatment with GSH, the emission intensity at 665 nm enhanced (Figure 1b), with the color of the solution changing to dark purple under visible light (Figure 1a) and the fluorescence quantum yield increasing up to 0.0315.

As for **BPO-DNP,** it revealed similar response to SO_2_ in absorption and emission spectra (Figures S1 and S2). However, there were almost no absorption or fluorescence changes when treated with GSH or other biothiols, respectively (Figures S1 and S3). It indicated that **BPO-DNP** could only detect SO_2_, while **BPO-DNSP** could detect SO_2_ in the green channel with a 105 nm Stokes shift, and biothiols in the near-infrared emission channel with an approximate 109 nm Stokes shift. The emission shift for the two channels was as high as 170 nm, which was favorable for fluorescence imaging in living cells. From the aforementioned results, we can infer that fluorophore caging with a sulfonate bond revealed better reactivity and sensitivity toward biothiols compared with an ether bond.

### 2.3. Optical Response of BPO-DNSP to SO_2_ and Biothiols

The optical properties of **BPO-DNSP** to detect SO_2_ and biothiols were inspected in detail. As shown in Figure S4, the fluorescent intensities of **BPO-DNSP** at 495 nm almost had no change in the pH range from 4.0 to 10.0. However, once 500 μM SO_2_ was added, the 495 nm fluorescence intensity increased dramatically when the pH increased from 4.0 to 8.0. A further increase in the pH to 10.0 led to a gradual decrease in fluorescence intensity. Therefore, pH 8.0 was chosen to be the optimal condition.

**Figure 2:**
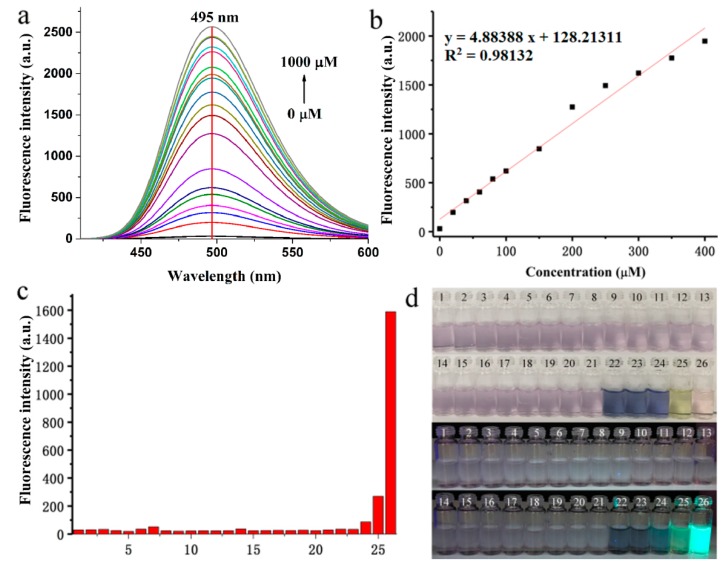
Fluorescence responses of **BPO-DNSP** to SO_2_. (**a**) Fluorescence responses of **BPO-DNSP** (10 μM) to different concentrations of SO_2_ (0–1000 μM); (**b**) Plot of **BPO-DNSP** fluorescence intensity to 0–400 μM SO_2_ at 495 nm; (**c**) fluorescence intensity and (**d**) color changes of **BPO-DNSP** (10 μM) treated with 500 μM of various relevant analytes. λ_ex_ = 390 nm. Slit: 2.5 nm/5 nm. 1. **BPO-DNSP**, 2. Ala, 3. Arg. 4. Asp, 5. Glu, 6. Gly, 7. His, 8. Ile, 9. Leu, 10. Lys, 11. Met, 12. Phe, 13. Pro, 14. Ser, 15. Val, 16. H_2_O_2_, 17. NaClO, 18. TBHP, 19. NO_3_^−^, 20. NO_2_^−^, 21. SO_4_^2−^, 22. Cys, 23. Hcy, 24. GSH, 25. H_2_S, 26. SO_2_.

Figure 2 showed that the addition of SO_2_ from 0 to 1000 μM induced the intensity of fluorescence at 495 nm to increase gradually (Figure 2a,b), demonstrating up to an 86-fold enhancement with/without SO_2_. The fluorescence intensity of the probe **BPO-DNSP** showed a linear relationship with SO_2_ concentration ranges from 0 to 400 μM. A 149.3 nM detection limit was observed. As shown in the kinetic studies (Figure S5), the fluorescence at 495 nm remained constant within about 2 h after a reaction with excess SO_2_. The selectivity of **BPO-DNSP** towards SO_2_ at 495 nm over other species was then evaluated. It was observed that SO_2_ showed a remarkable fluorescence enhancement, while other biological molecules such as amino acids (Ala, Arg, Asp, Glu, Gly, His, Ile, Leu, Lys, Met, Phe, Pro, Ser, Val), reactive oxygen species (NaClO, H_2_O_2_, TBHP), reactive nitrogen species (NO_2_^−^ and NO_3_^−^), and other reactive sulfur species (SO_4_^2−^, H_2_S, Cys, HCy, and GSH) led to negligible fluorescence changes. In addition, only SO_2_ induced a strong green fluorescence enhancement under 365 nm UV light, as shown in Figure 2d.

The 665 nm fluorescence intensity of **BPO-DNSP** showed to be almost constant, with pH increasing from 4.0 to 10.0 (Figure S6). Once 500 μM GSH was added, the fluorescence intensity firstly increased and reached a maximum at pH 6.0, then decreased with continuous increase in pH. Therefore, pH 6.0 should be the optimal pH value for detecting biothiols in the near-infrared region. As we know, biothiols were widespread in living cells with a concentration in the millimolar range [2,9], while the concentration of SO_2_ was much lower than that. With the aim to detect intracellular SO_2_ and biothiols with **BPO-DNSP** under the same conditions, pH 8.0 was chosen as the detection condition for both SO_2_ and biothiols.

**Figure 3:**
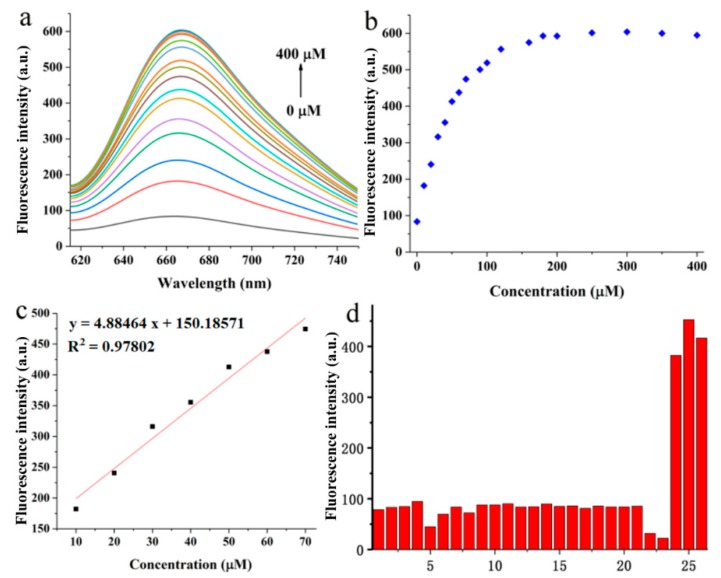
(**a**) Fluorescence responses of **BPO-DNSP** to GSH and (**b**) fluorescence responses of **BPO-DNSP** (10 μM) to different concentrations of GSH (0–400 μM); (**c**) plot of **BPO-DNSP** fluorescence intensity to 10–70 μM GSH at 665 nm; (**d**) fluorescence intensity changes of **BPO-DNSP** (10 μM) treated with 500 μM of various relevant analytes. λ_ex_ = 556 nm. Slit: 10 nm/10 nm. 1. **BPO-DNSP**, 2. Ala, 3. Arg. 4. Asp, 5. Glu, 6. Gly, 7. His, 8. Ile, 9. Leu, 10. Lys, 11. Met, 12. Phe, 13. Pro, 14. Ser, 15. Val, 16. H_2_O_2_, 17. NaClO, 18. TBHP, 19. NO_3_^−^, 20. NO_2_^−^, 21. SO_4_^2−^, 22. H_2_S, 23. SO_2_, 24. Cys, 25. Hcy, 26. GSH.

When GSH concentration increased from 0 to 200 μM, the 665-nm fluorescence intensity increased gradually as shown in Figure 3a. The intensity enhancement factor was 7.22, and the detection limit was 276.8 nM (Figure 3b). Kinetic studies (Figure S7) demonstrated that the fluorescence of **BPO-DNSP** reached the maximum within 15 min when reacted with 500 μM GSH. **BPO-DNSP** also exhibited good selectivity for biothiols at 665 nm (Figure 3d and Figure S8), which displayed no increase in other biological molecules (Ala, Arg, Asp, Glu, Gly, His, Ile, Leu, Lys, Met, Phe, Pro, Ser, Val, NaClO, H_2_O_2_, TBHP, NO_2_^−^ and NO_3_^−^, SO_4_^2−^, H_2_S, SO_2_). Furthermore, only biothiols induced a color change from purple to dark purple under visible light (Figure 2d).

### 2.4. Optical Response of BPO-DNP to SO_2_

**Figure 4:**
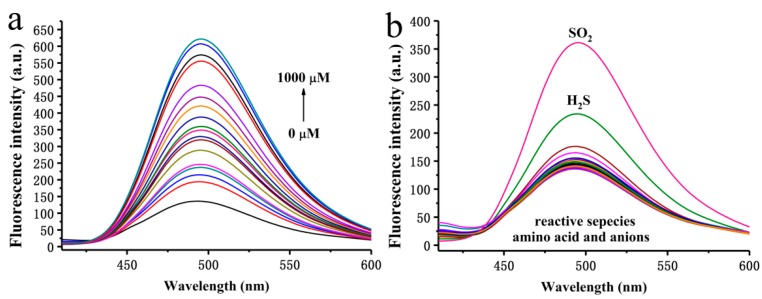
Fluorescence responses of **BPO-DNP** to SO_2_. (**a**) Fluorescence responses of **BPO-DNP** (10 μM) to different concentrations of SO_2_ (0–1000 μM). (**b**) Fluorescence response of **BPO-DNP** (10 μM) with 500 μM of different kinds of amino acids (Arg, Met, Ser, Asp, Gly, Ala, His, Val, Lys, Leu, Glu, Pro, Ile, Phe), reactive species, and anions (H_2_O_2_, NaClO, TBHP, NO_3_^−^, NO_2_^−^, SO_4_^2−^, Cys, Hcy, GSH, H_2_S, SO_2_) in 10 mM pH 8.0 phosphated-buffered saline (PBS) and dimethylsulfoxide (DMSO) mixture solution (8:2, *v*/*v*), λ_ex_ = 556 nm. Slit: 10 nm/10 nm.

With the excitation at 390 nm, **BPO-DNP** (10 μM) showed a gradual increase following the addition of SO_2_ with a concentration from 0 to 1000 μM (Figure 4a), with an intensity enhancement factor of 4.6. The fluorescence quantum yield of **BPO-DNP** increased from 0.009 to 0.038. An excellent linearity between the fluorescence intensity and SO_2_ concentration (20–200 μM) could be observed (Figure S9), and the detection limit was calculated to be 1.58 μM. The selectivity of **BPO-DNP** to SO_2_ over other species was investigated in Figure 4b. Nevertheless, other than SO_2_, H_2_S also evidently enhanced the fluorescence intensity, which implied a bad selectivity of **BPO-DNP** towards SO_2_ (Figure 4b).

### 2.5. Fluorescence Imaging of SO_2_ and Biothiols in Living Cells

The applicability of **BPO-DNSP** for intracellular imaging of SO_2_ and biothiols was evaluated with HeLa cells. First, a standard cell counting kit-8 (CCK-8) assay was conducted with HeLa cells at different concentrations (Figures S10 and S11). Compared to **BPO-DNP**, **BPO-DNSP** exhibited more favorable biocompatibility. More than 85% of the cells survived when incubated with 10 μM **BPO-DNSP** for 24 h, suggesting that the probe could be used for imaging applications in living cells. In contrast, only about 65% of the cells survived when treated with 10 μM **BPO-DNP** for 24 h.

**Figure 5:**
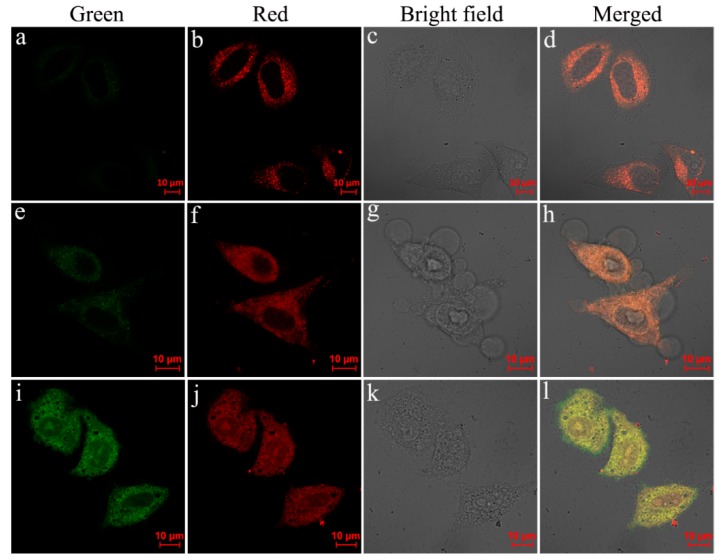
Confocal fluorescence images of living HeLa cells (**a**–**d**) incubated with 10 μM **BPO-DNSP**; (**e**–**h**) HeLa cells pretreated with 50 μM NEM and further incubated with 10 μM **BPO-DNSP**; (**i**–**l**) after pretreatment with 50 μM NEM, HeLa cells were loaded with 10 μM **BPO-DNSP** and then incubated with SO_2_ (500 μM) in the green channel, red channel, bright-field, and merged images. For green channel: λ_ex_ = 405 nm, λ_em_ = 460–530 nm; for red channel: λ_ex_ = 560 nm, λ_em_ = 620–720 nm.

**Figure 6:**
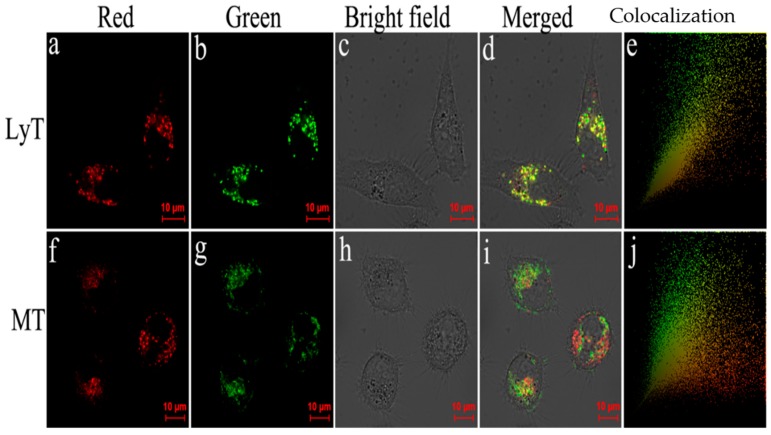
Colocalization experiments of **BPO-DNSP** with LyT (**a**–**e**) and MT (**f**–**j**) in HeLa cells. (**a**) and (**f**) fluorescence image of **BPO-DNSP** (10 μM) on red channel (λ_em_ = 620–720 nm) with excitation at 560 nm; fluorescence image of 1 μM LyT (**b**) and MT (**g**) on green channel (λ_em_ = 510–600 nm) with excitation at 488 nm; (**c**) and (**h**) image from bright field; (**d**) and (**i**) merged image of red, green, and bright field channels, colocalization of **BPO-DNSP** with (**e**) lysosome tracking (LyT) (**j**) and mitochondrial targeting (MT).

Colocalization experiments were then performed to confirm the lysosome-specific capability of **BPO-DNSP**. When the HeLa cells were incubated with **BPO-DNSP** at 37 °C for 30 min, a strong emission in the red channel was observed, indicating that **BPO-DNSP** could detect intracellular biothiols (detailed result can be found in Figure 5). Then, the HeLa cells were incubated with **BPO-DNSP** and commercial lysosome targeting (LyT, Figure 6a–e and Figure S12) or mitochondrial-targeting (MT, Figure 6f–j and Figure S13), green, fluorescent probes. The green fluorescence was emitted from Lyt or MT, and red fluorescence was ascribed to **BPO-DNSP**. The Pearson’s correlation coefficient of red fluorescence with Lyt was 0.87 and 0.57 for MT, indicating that **BPO-DNSP** could specifically target lysosomes in living HeLa cells.

Ultimately, fluorescence imaging of biothiol and SO_2_ was explored in living cells. As shown in Figure 5, HeLa cells with **BPO-DNSP** that were incubated for 30 min at 37 °C displayed almost no fluorescence in the green channel (Figure 5a), but they showed strong fluorescence in the red channel (Figure 5b). When the cells were pretreated with NEM (Figure 5e–h) for 30 min and further incubated with **BPO-DNSP** for another 30 min, HeLa cells exhibited a significant decrease in fluorescence in the red channel (Figure 6f) under the same image acquisition, indicating that **BPO-DNSP** could detect intracellular biothiols. At the same time, the green fluorescence ascribed to SO_2_ (Figure 5e) increased in contrast with Figure 5a, which demonstrates the potential to monitor endogenous SO_2_ production and the conversion from biothiols to SO_2_ without adding exogenous enzymes. If the cells were further treated with 500 μM exogenous SO_2_ for another 30 min (Figure 5i–l), the fluorescence in the green channel (Figure 5i) dramatically increased. These results indicated that **BPO-DNSP** could not only be used to image intracellular SO_2_ and biothiols from two different emission channels, but also provide a possible way to monitor the production of endogenous SO_2_ and the conversion from biothiols to SO_2_ without adding exogenous enzymes to living cells.

## 3. Experimental

### 3.1. Materials and Instruments

All chemical reagents were purchased from Energy Chemical and used without further purification. Na_2_SO_3_ was used as the source of SO_2_. All solvents for optical spectroscopic studies were high-pressure, liquid-chromatography grade. Thin-layer chromatography (TLC) analyses were performed on silica gel GF 254, while column chromatographic purifications were conducted over silica gel (300–400 mesh). UV-visible absorption spectra were obtained with a Hitachi PharmaSpec UV-1900 UV-visible spectrophotometer. A Hitachi F7000 spectrofluorometer was utilized to acquire the fluorescence spectra. A Bruker Daltonics Bio time-of-flight mass spectrometer was employed for high-resolution mass spectra. ^1^H NMR (600 MHz) and ^13^C NMR (150 MHz) spectra were recorded on a Bruker AMX-600 using tetramethyl silane (TMS) as the internal reference. Confocal fluorescent images were recorded with a Zeiss LSM 780 confocal laser scanning microscope.

### 3.2. Synthesis of BPO-DNSP and BPO-DNP

**BPOH** was synthesized according to the literature [51]. **BPO-DNSP**: 96 mg **BPOH** (0.2 mmol), 100 mg Et_3_N (1.0 mmol), and 10 mL dichloromethane were added to a 25-mL round-bottom flask. 64 mg (0.24 mmol) 2,4-dinitrobenzenesulfonyl chloride in 2 mL dichloromethane were added dropwise to the above mixture at 0 °C with vigorous stirring. The mixture was further stirred at 0 °C for 0.5 h and at room temperature for another 3 h. Then, 50 mL of DCM was added to the reaction mixture, and the mixture was washed with 50 mL water for three times. The organic layer was collected and dried with sodium sulfate. Subsequently, the solvent was removed under reduced pressure in a rotary evaporator. The crude product was purified via silica-gel column chromatography with DCM to DCM/MeOH (20/1, *v*/*v*) to obtain 52 mg dark purple solid (32% yield). ^1^H NMR (600 MHz, CDCl_3_), 8.86 (d, 1H, *J* = 1.8 Hz), 8.50 (dd, 1H, *J* = 2.4 Hz, *J* = 9.0 Hz), 8.22 (d, 1H, *J* = 8.4 Hz), 7.95 (d, 1H, *J* = 7.8 Hz), 7.65 (t, 1H, *J* = 7.2 Hz), 7.56 (t, 1H, *J* = 7.2 Hz), 7.37 (d, 2H, *J* = 8.4 Hz), 7.31 (s, 1H), 7.21 (t, 3H, *J* = 8.4 Hz), 6.48 (d, 2H, *J* = 9.0 Hz), 6.38 (d, 1H, *J* = 2.4 Hz), 6.35 (dd, 1H, *J* = 2.4 Hz, *J* = 9.0 Hz), 3.35 (q, 4H, *J* = 7.2 Hz), 2.73–2.70 (m, 1H), 2.57–2.53 (m, 1H), 2.05–2.03 (m, 1H), 1.65–1.60 (m, 3H), 1.18 (t, 6H, *J* = 7.2 Hz). ^13^C NMR (125 MHz, CDCl_3_), 170.0, 152.3, 152.2, 150.9, 149.4, 149.0, 147.3, 146.5, 137.5, 134.6, 134.0, 133.7, 132.0, 131.2, 129.4, 128.6, 127.6, 126.4, 125.0, 123.5, 123.3, 121.8, 120.3, 109.0, 108.9, 104.7, 97.1, 44.4, 27.1, 23.0, 22.3, 12.6. HRMS (ESI): *m*/*z* [M]^+^ calculated for C_37_H_32_N_3_O_10_S^+^: 710.1803; found 710.1798.

**BPO-DNP**: 96 mg (0.2 mmol) **BPOH** was dissolved in 5 mL dimethyl formamide. Then, 44.6 mg (0.24 mmol) 2,4-dinitrofluorobenzene and 110 mg K_2_CO_3_ (0.8 mmol) were added respectively. After being stirred for 5 h at 50 °C, the solvent was removed under reduced pressure in a rotary evaporator. The crude product was purified via silica-gel column chromatography with DCM to DCM/MeOH (20/1, *v*/*v*) to obtain 71.6 mg dark purple solid (48% yield). ^1^H NMR (600 MHz, CDCl_3_), 8.86 (d, 1H, *J* = 2.4 Hz), 8.34 (dd, 1H, *J* = 2.4 Hz, *J* = 9.0 Hz), 7.96 (d, 1H, *J* = 7.8 Hz), 7.66 (t, 1H, *J* = 7.2 Hz), 7.56 (t, 1H, *J* = 7.2 Hz), 7.50 (d, 2H, *J* = 8.4 Hz), 7.38 (s, 1H), 7.24 (t, 1H, *J* = 7.8 Hz), 7.15 (d, 2H, *J* = 8.4 Hz), 7.11 (d, 1H, *J* = 9.0 Hz), 6.49 (d, 1H, *J* = 8.4 Hz), 6.42 (d, 1H, *J* = 1.8 Hz), 6.36 (dd, 1H, *J* = 2.4 Hz, *J* = 9.0 Hz), 3.37 (q, 4H, *J* = 7.2 Hz), 2.82–2.79 (m, 1H), 2.65–2.62 (m, 1H), 2.08–2.05 (m, 1H), 1.71–1.62 (m, 3H), 1.18 (t, 6H, *J* = 7.2 Hz). ^13^C NMR (125 MHz, CDCl_3_), 170.0, 156.1, 152.5, 152.2, 152.1, 149.4, 146.6, 141.5, 139.6, 135.9, 134.6, 131.7, 131.6, 129.3, 128.9, 128.6, 127.6, 125.0, 123.5, 123.4, 122.2, 120.3, 118.6, 108.8, 108.6, 104.5, 97.2, 44.4, 27.2, 23.0, 22.4, 12.6. HRMS (ESI): *m*/*z* [M]^+^ calculated for C_37_H_32_N_3_O_8_^+^: 646.2184; found 646.2185.

### 3.3. Absorption and Fluorescence Spectra Measurement

All the test solutions were conducted in 3.0 mL of PBS buffer/DMSO (8:2, *v*/*v*) solution by adding an aliquot of stock solution and 15 μL of the probe stock solution. Absorption and fluorescence spectra were monitored on a UV-visible spectrophotometer or fluorescent spectrofluorometer after incubating the test solution for 2 h for SO_2_ or 30 min for biothiols at room temperature.

### 3.4. Detection Limit

The detection limit was calculated based on the fluorescence titration with the following equation:

detection limit = 3 σ_bi_/m (1)

where σ_bi_ is the standard deviation of blank measurements and m is the slope between intensity versus sample concentration (signal-to-noise ratio of 3:1). The standard deviation of blank measurements was determined when the emission intensity of probe (10 μM) without Na_2_SO_3_ (at 495 nm) or GSH (at 665 nm) was measured ten times. Then, fluorescent titrations were conducted under the present conditions. A good linear relationship between the fluorescence intensity and the concentration of Na_2_SO_3_ or GSH was obtained. The slope between intensity versus Na_2_SO_3_ or GSH concentration was m in the aforementioned formula.

### 3.5. CCK-8 Assay for the Cell Cytotoxicity

HeLa cells were seeded into 96-well plates (about 7000 cells per well) and cultured overnight to a 70–80% cell confluence. After replacing the medium with 100 μL fresh medium, different concentrations of **BPO-DNSP** and **BPO-DNP** (in DMSO stock solution) were then added and incubated for 24 h. Then, 10-μL CCK-8 mixed in 90 μL PBS was added to each well for additional 1 h incubation. The absorbance was measured at a wavelength of 450 nm. The viability of the treated cells was determined relative to the cell treated with same amount of DMSO as 100% activity.

### 3.6. Cell Culture and Confocal Imaging

HeLa cells were cultured in DMEM (Dullbecco Modified Eagle Medium) containing 10% fetal bovine serum (FBS) and 1% penicillin–streptomycin in a 5% CO_2_/95% air incubator at 37 °C. For colocalization and fluorescent imaging experiments, cells (4 × 10^3^ well^−1^) were placed on glass-bottom dishes and incubated for 24 h. Before staining, the cells were washed three times with physiological PBS.

For colocalization experiments with Lyso Tracker Green (Lyt) or Mito Tracker Green (MT), HeLa cells were incubated with **BPO-DNSP** (10 µM) in PBS for 30 min at 37 °C. After removing the medium and washing them three times with PBS, the cells were incubated with 1.0 µM Lyt or MT for another 30 min. Finally, cells were washed with PBS for fluorescent imaging. The fluorescence of LyT or MT in the green channel were collected at 510–600 nm with excitation at 488 nm, and the fluorescence of **BPO-DNSP** in the red channel was collected at 620–720 nm with excitation at 560 nm. Image Pro Plus 6.0 software was used for the colocalization analysis by measuring the colocalization of the merged figure in the red and green channels to create calculated correlations between the images for the Pearson correlation coefficient and to create a color view of color colocalization.

For living cell imaging of SO_2_ and biothiols experiments: the Hela cells were incubated with **BPO-DNSP** (10 µM) in PBS for 30 min at 37 °C as the control experiment. For the thiol-blocking experiment, Hela cells were pretreated with 50 μM NEM for 30 min at 37 °C and then incubated with **BPO-DNSP** (10 µM) for another 30 min. For fluorescent imaging of SO_2_ in living cells, after pretreatment with 50 μM NEM for 30 min at 37 °C, Hela cells were incubated with **BPO-DNSP** (10 µM) for another 30 min and then incubating with 500 μM SO_2_. The fluorescence for the green channel was recorded at the wavelength range 460–530 nm with excitation at 405 nm. For the red channel, the confocal fluorescent images were collected over the range 620–720 nm with excitation at 560 nm.

## 4. Conclusions

In summary, two fluorescent probes caged with 2,4-dinitrobenzenesulfonyl (**BPO-DNSP**) and 2,4-dinitrofluorobenzene (**BPO-DNP**) for sensing SO_2_ and biothiols from two different emission channels in living cells were developed. **BPO-DNP** can only detect SO_2_ with low sensitivity and poor selectivity. However, **BPO-DNSP** can sensitively and selectively respond to SO_2_ and emit green fluorescence with a large Stokes shift over 105 nm, and it can react with biothiols in the near-infrared emission channel with a Stokes shift over 109 nm. The emission wavelength shift from those two channels was as high as 170 nm. Colocalization experiments demonstrated that **BPO-DNSP** possessed lysosome-targeting properties. Furthermore, **BPO-DNSP** can not only be used for imaging intracellular SO_2_ and biothiols, but can also be used for monitoring the conversion from biothiols to SO_2_ without adding exogenous enzymes, which can help us better understand the physiological nature of RSS. In comparison with the reported probes [24,25,26,27,28] for two-channel imaging of SO_2_ and biothiols, **BPO-DNSP** exhibited three advantages for bioimaging: (1) as far as 170 nm emission shift from two different channels; (2) near-infrared wavelength detection of intracellular biothiols; and (3) monitoring the conversion from biothiols to SO_2_ without adding exogenous enzymes. Moreover, **BPO-DNSP** revealed its superiority in biological applications as it avoids potential interference compared with reported probes using an aldehyde group as a reaction site. However, **BPO-DNSP** also is disadvantageous due to its slow kinetics for both SO_2_ and biothiols. Further modification with much stronger electron-withdrawing groups to replace oxonium salt and the caging group is necessary for improving the reactivity and kinetics.

## Supplementary Materials

The following are available online, Figures S1–S12: Optical Properties; Figures S13–S18: ^1^H NMR, ^13^C NMR, and HRMS spectrum.
