# Supplementary material for: Lysosome-Targeted Single Fluorescence Probe for Two-Channel Imaging Intracellular SO2 and Biothiols
Source: Molecules. 2019 Feb 11;24(3):618. doi: 10.3390/molecules24030618 (PMC6384543; doi:10.3390/molecules24030618)
Supplement: Supplementary file 1 [file molecules-24-00618-s001.pdf]

Supporting information

Lysosome-targeted single fluorescence probe for two-channel imaging intracellular SO_2 and biothiols

Yue Wang, Li Liu, Xian-Li Zhou, Ming-Yu Wu*

School of Life Science and Engineering, Southwest Jiaotong University, Chengdu 610031, China.

* Corresponding author： E-mail: wumy1050hx@swjtu.edu.cn (M.-Y.W).

Table of contents

1. **Optical Properties** **S3-S12**

2. **^{1}H NMR, ^{13}C NMR, and HRMS spectrum** **S13-S18**

1. Optical Properties

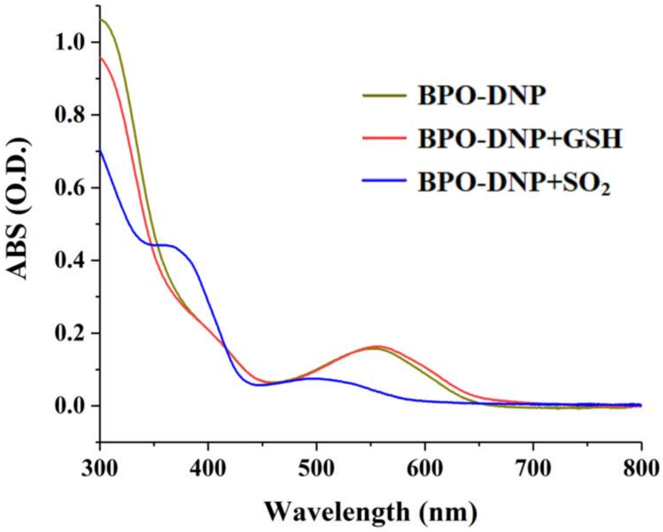

Fig. S1. UV-Vis absorption spectrum of **BPO-DNP** (10 μM) and its interacting with 500 μM SO_2 or GSH

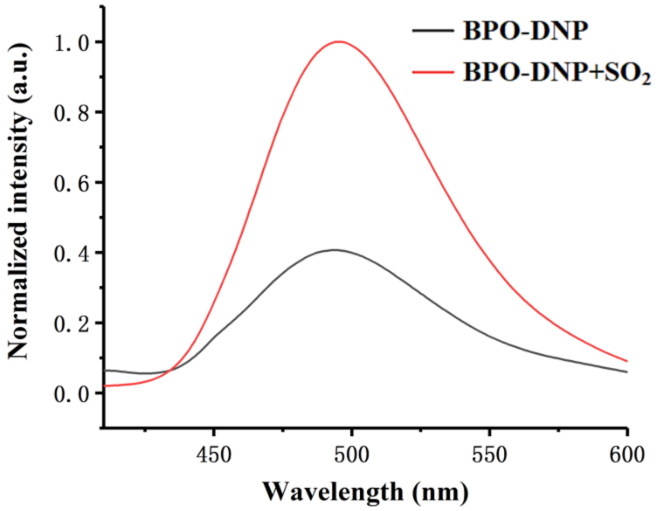

Fig. S2. Normalized fluorescence spectrum of **BPO-DNP** (10 μM) and its interacting with 500 μM SO_2

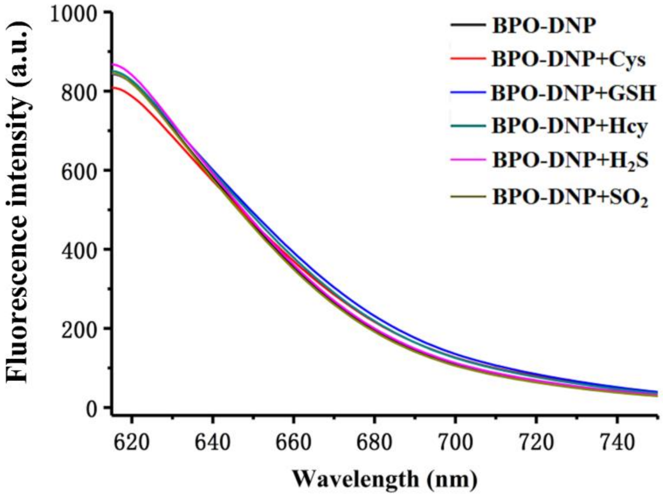

Fig. S3. Fluorescent spectra of **BPO-DNP** (10 μM) and its interacting with 500 μM different kinds of RSS in near-infrared region. λ_{ex} = 556 nm. Slit: 10 nm/10 nm.

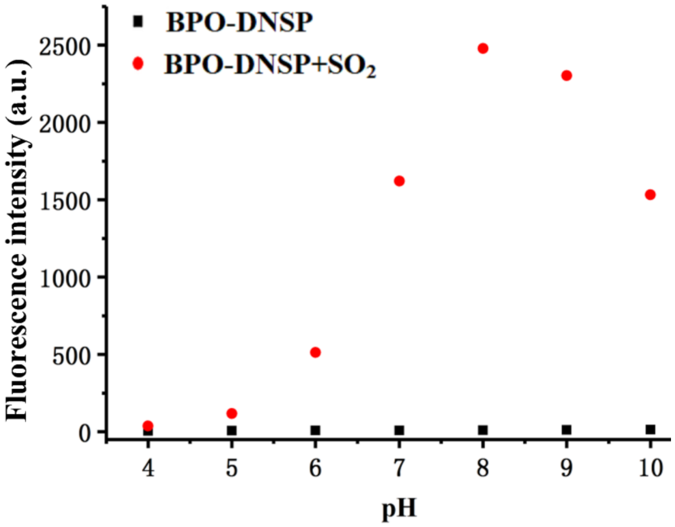

Fig. S4. pH dependent fluorescent intensity of **BPO-DNSP** (10 μM) and its interacting with SO_2 (500 μM) at 495 nm. λ_{ex} = 390 nm, λ_{em} = 495 nm, Slit: 2.5 nm/5 nm

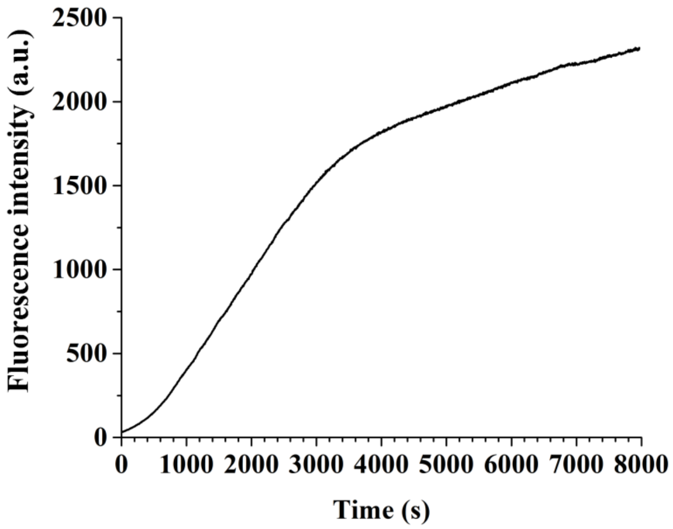

Fig. S5. Kinetic studies of **BPO-DNSP** (10 μM) with SO_2 (1000 μM). λ_{ex} = 390 nm, λ_{em} = 495 nm, Slit: 2.5 nm/5 nm.

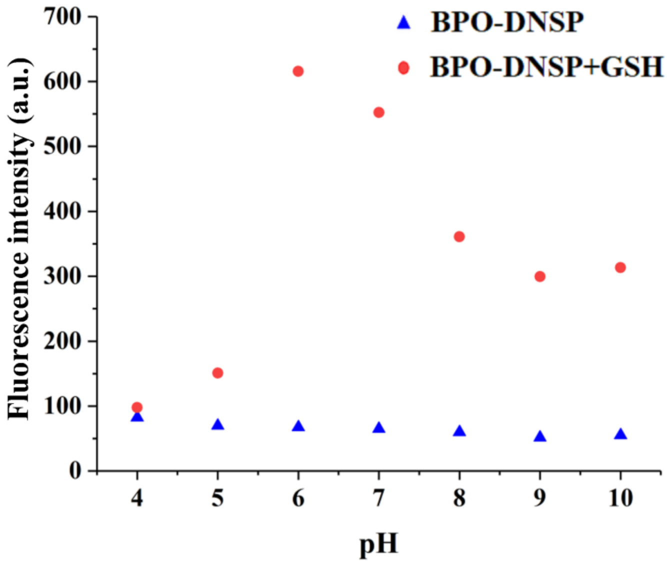

Fig. S6. pH dependent fluorescent intensity of **BPO-DNSP** (10 μM) and its interacting with GSH (500 μM) at 665 nm. λ_{ex} = 556 nm, λ_{em} = 665 nm, Slit: 10 nm/10 nm

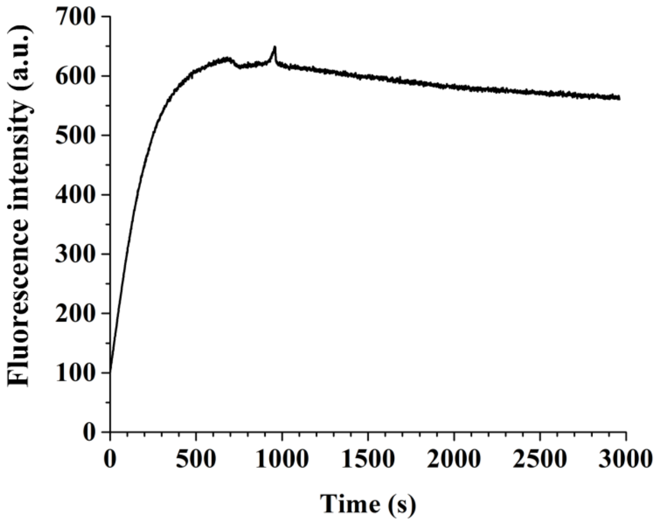

Fig. S7. Kinetic studies of **BPO-DNSP** (10 μM) with GSH (500 μM). λ_{ex} = 556 nm, λ_{em} = 665 nm, Slit: 10 nm/10 nm.

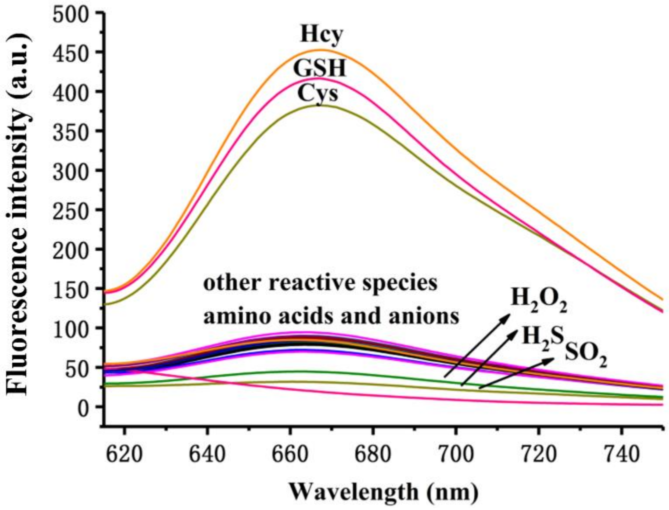

Fig. S8. Fluorescence response of **BPO-DNSP** (10 μM) with 500 μM different kinds of biology species (Arg, Met, Ser, Asp, Gly, Ala, His, Val, Lys, Leu, Glu, Pro, Ile, Phe, H_2O_2, NaClO, TBHP, NO_3^-, NO_2^-, SO_4^{2-}, Cys, Hcy, GSH, H_2S, SO_2) in 10 mM pH 8.0 PBS buffer and DMSO mixture solution (8:2, v/v), λ_{ex} = 556 nm. Slit: 10 nm/10 nm.

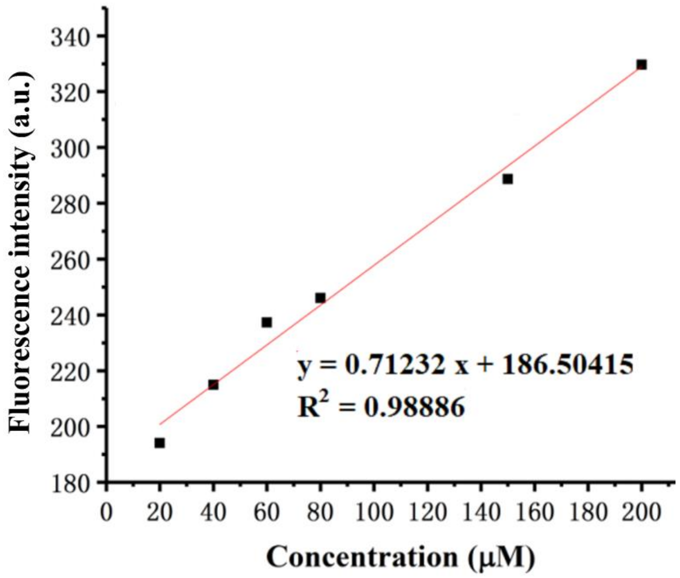

Fig. S9. (a) Fluorescence responses of **BPO-DNP** (10 μM) to different concentrations of SO_2 (0-1000 μM) (λ_{ex} = 390 nm, Slit: 5 nm/5 nm) in a 10 mM PBS:DMSO = 8:2 pH 8.0 buffer solution. (b) Plot of **BPO-DNP** fluorescence intensity to 20-200 μM Na_2SO_3 at 495 nm.

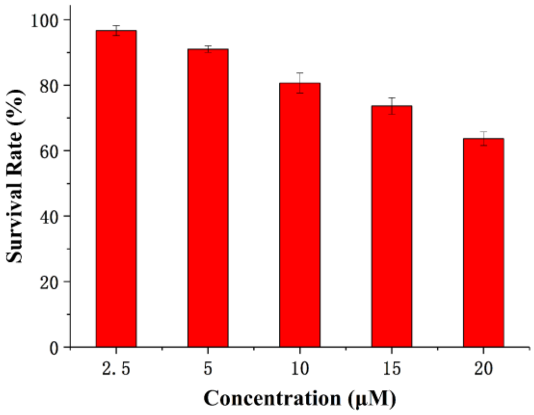

Fig. S10. Cell viability of HeLa cells stained with different concentrations of **BPO-DNSP.**

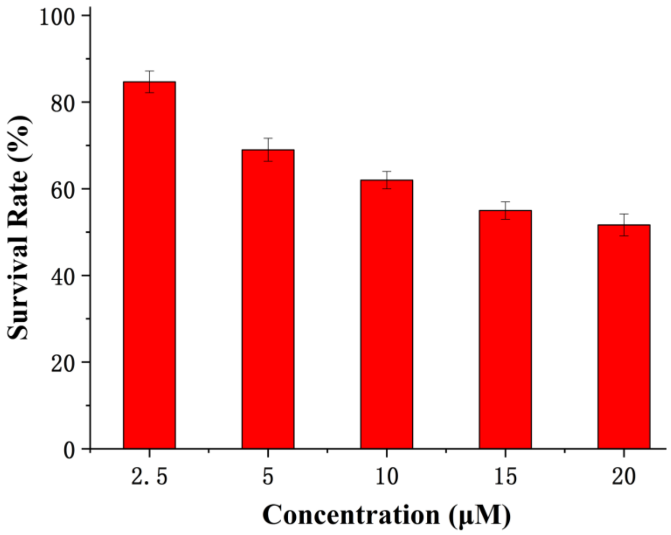

Fig. S11. Cell viability of HeLa cells stained with different concentrations of **BPO-DNP.**

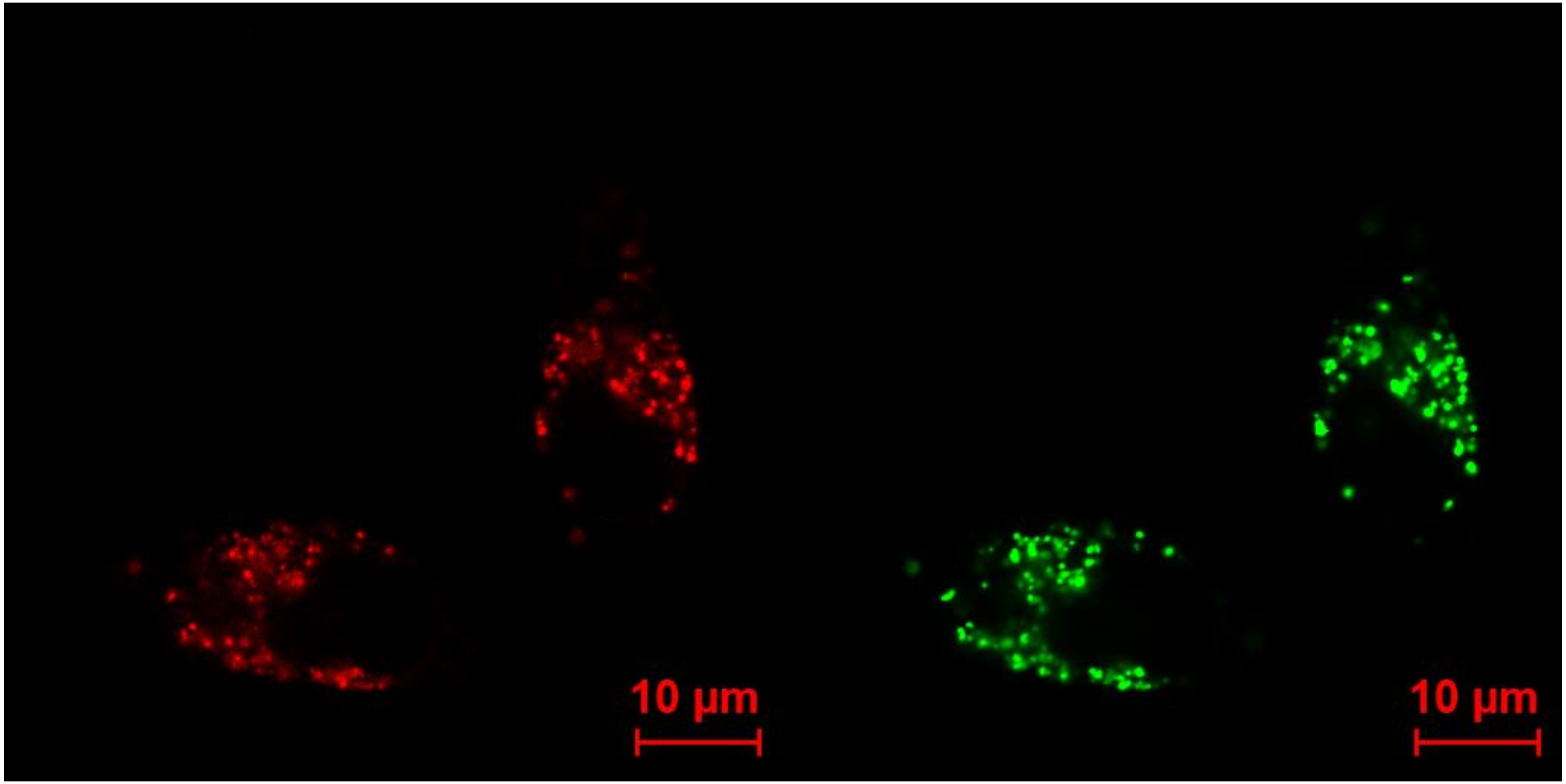
10 µm
10 µm

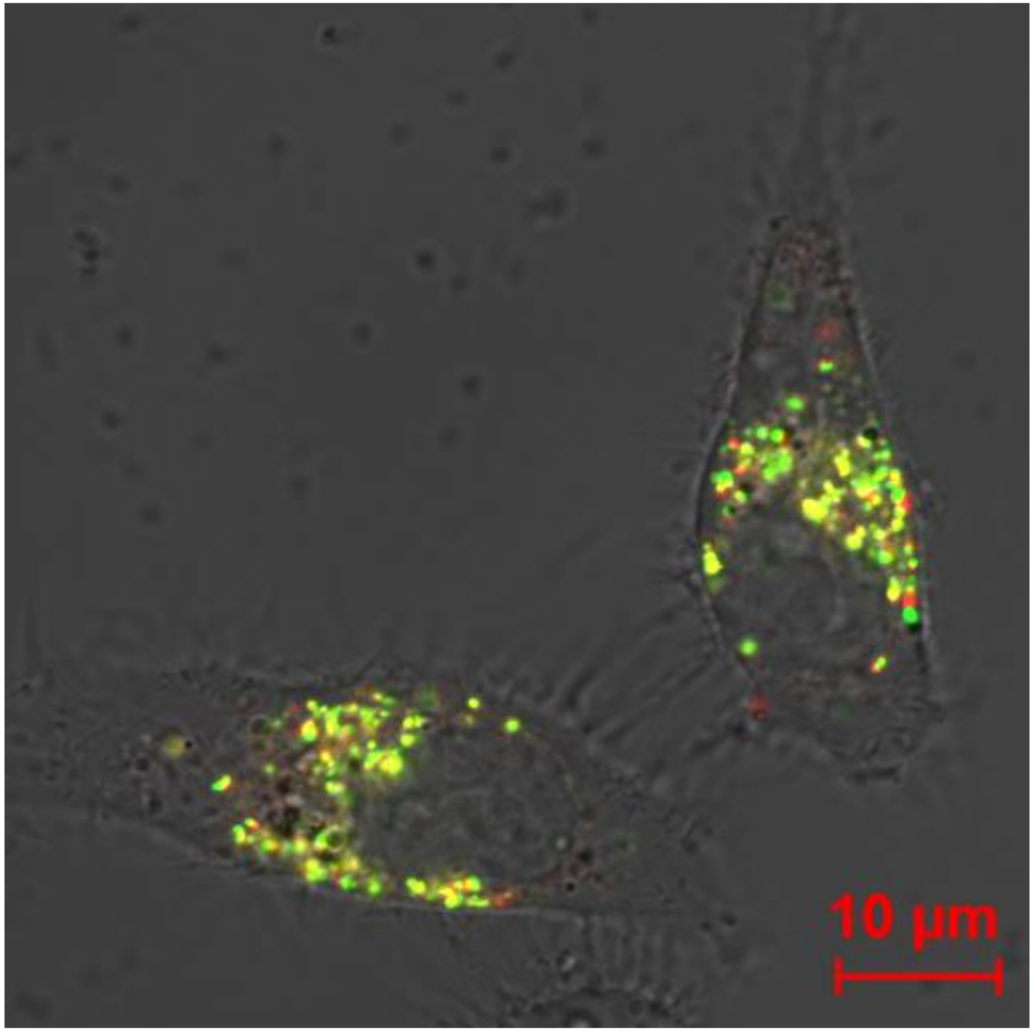

Fig. S12. Magnification of Figure 5a-d for lysosome co-localization experiment.

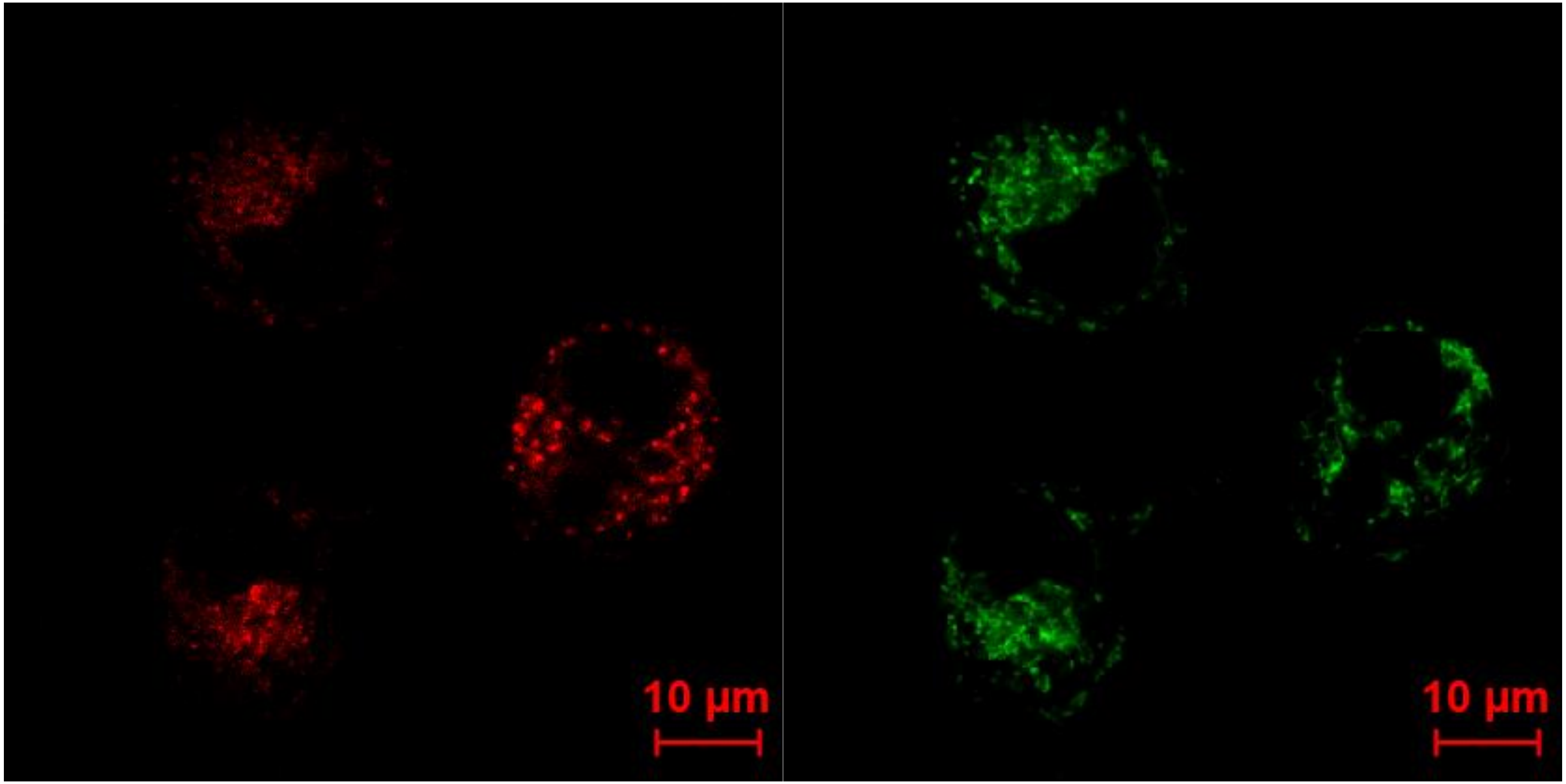
10 µm
10 µm

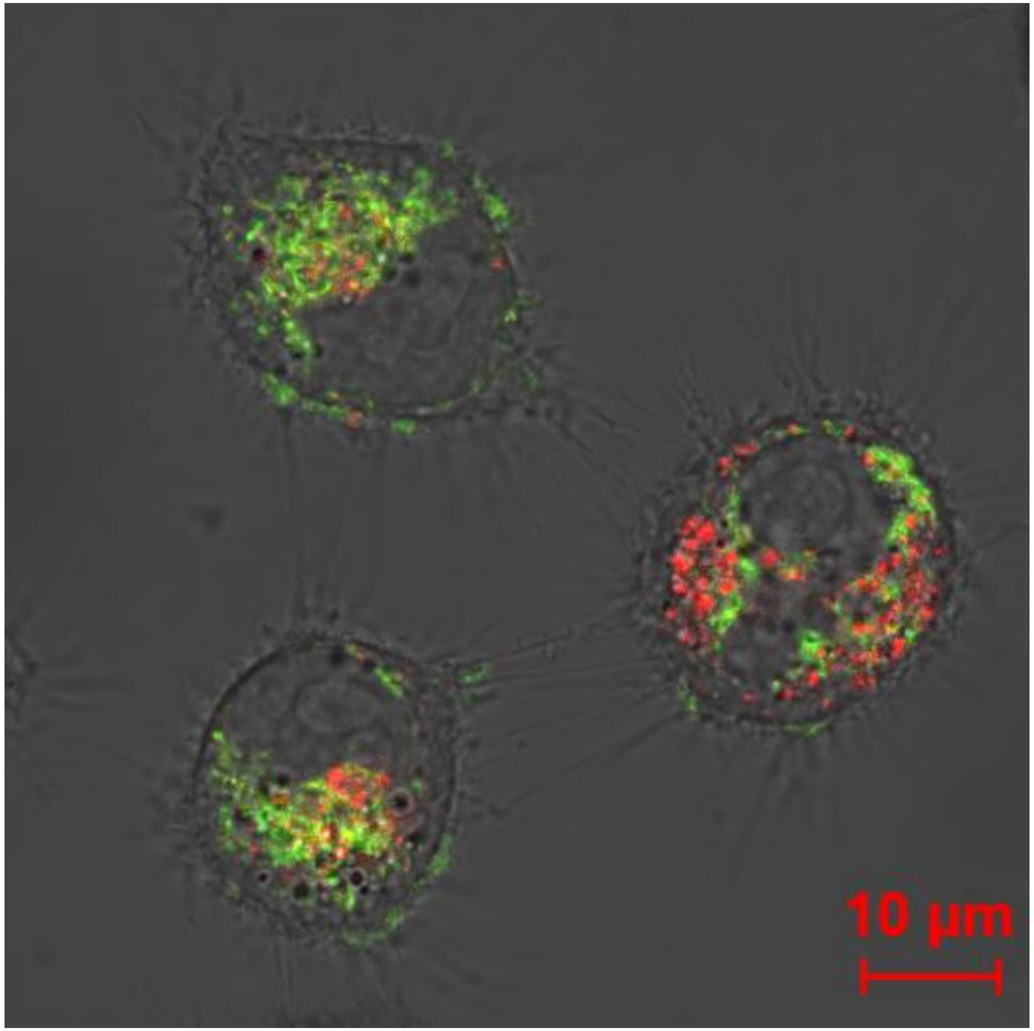

Fig. S13. Magnification of Figure 5a-d for mitochondrial co-localization experiment.

2. ^{1}H NMR, ^{13}C NMR, and HRMS spectrum

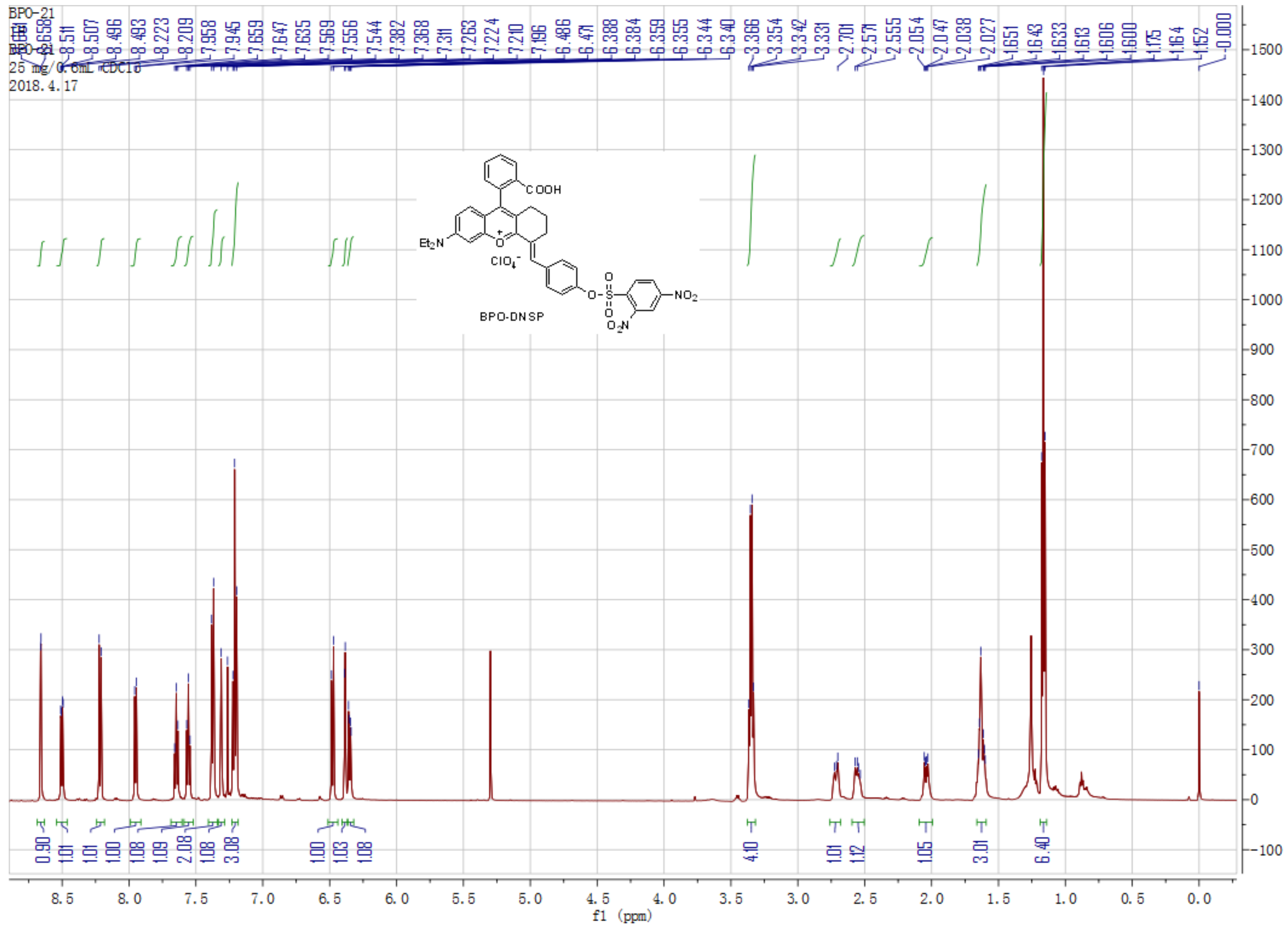

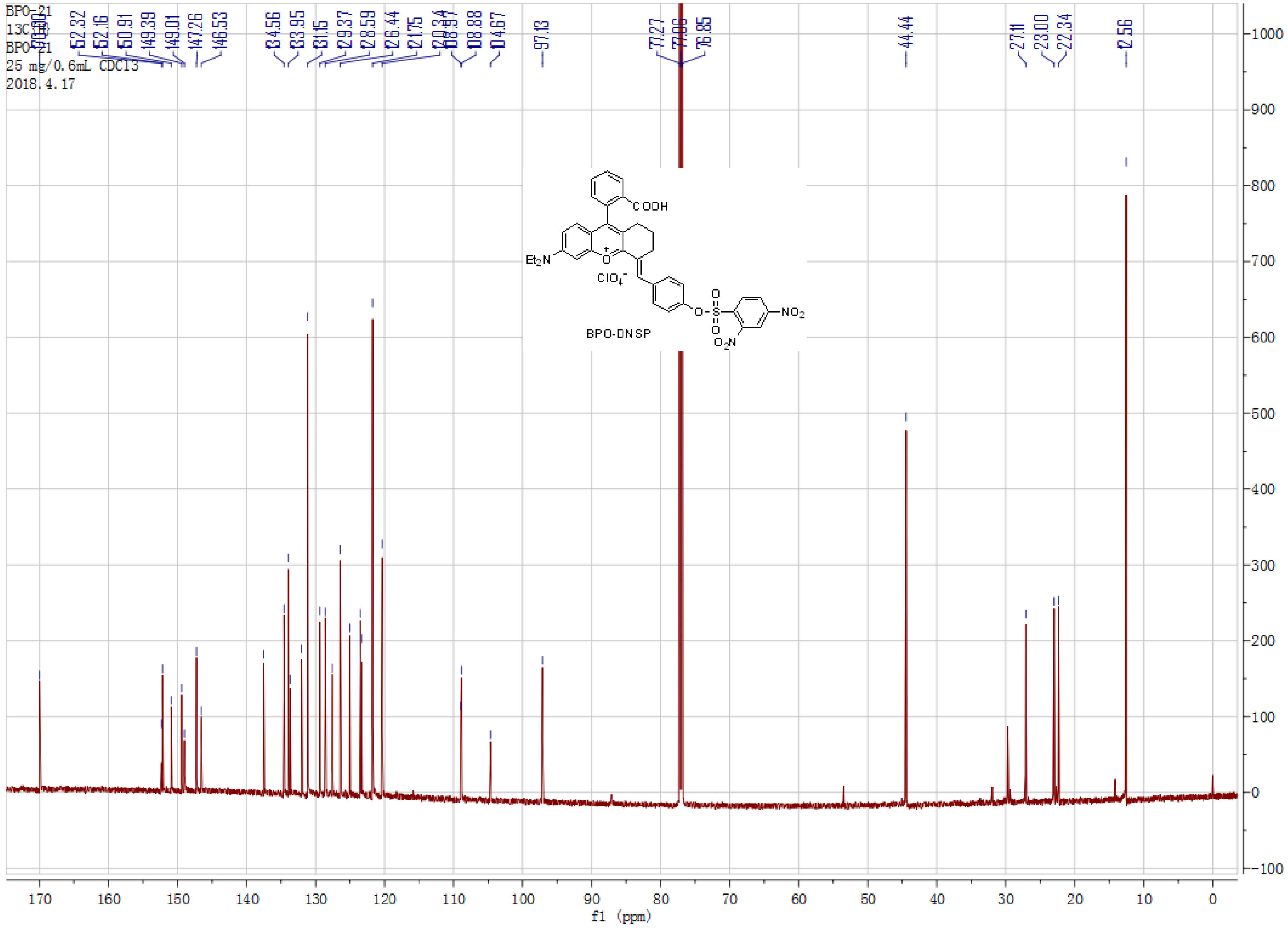

BPO-21
BPO-21
25 mg/0.6mL CDCl3
2018.4.17
152.32
152.16
150.91
149.39
149.01
147.26
146.53
134.56
133.95
131.15
129.37
128.59
126.44
121.75
108.88
104.67
97.13
77.27
76.85
44.44
27.11
23.00
22.34
12.56
COOH
Et2N
ClO4-
NO2
O2N
BPO-DNSP
f1 (ppm)

BPO-DNSP

MS(E+)

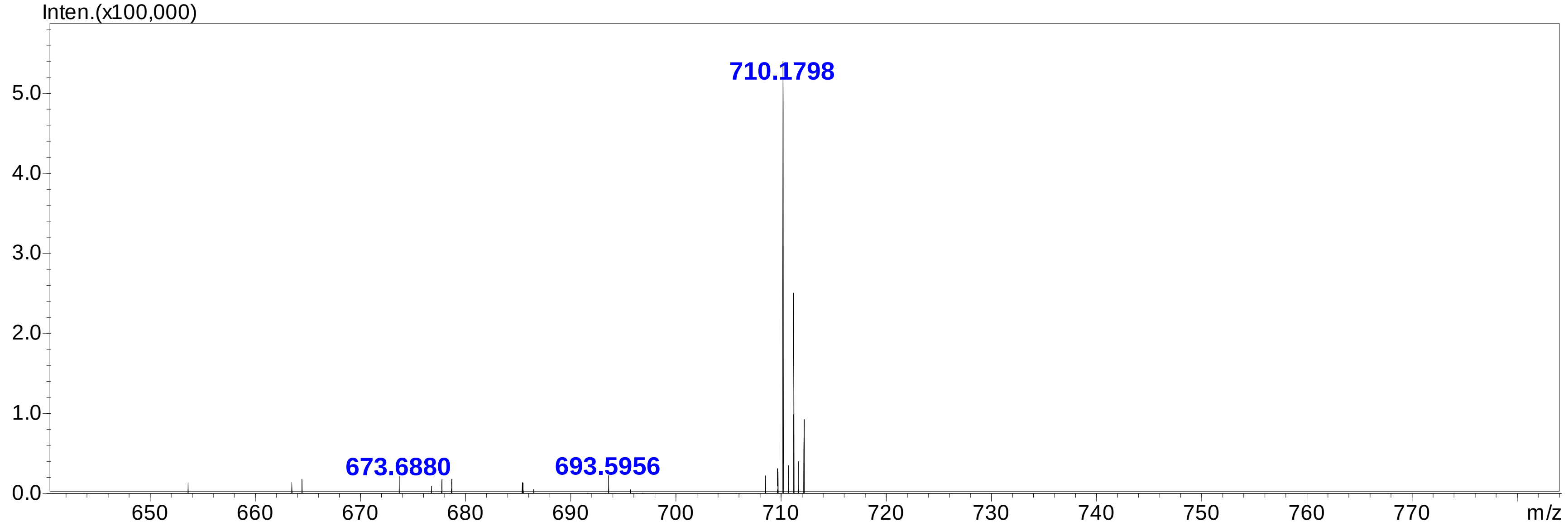
Inten.(x100,000)
5.0
4.0
3.0
2.0
1.0
0.0
710.1798
673.6880
693.5956
650
660
670
680
690
700
710
720
730
740
750
760
770
m/z

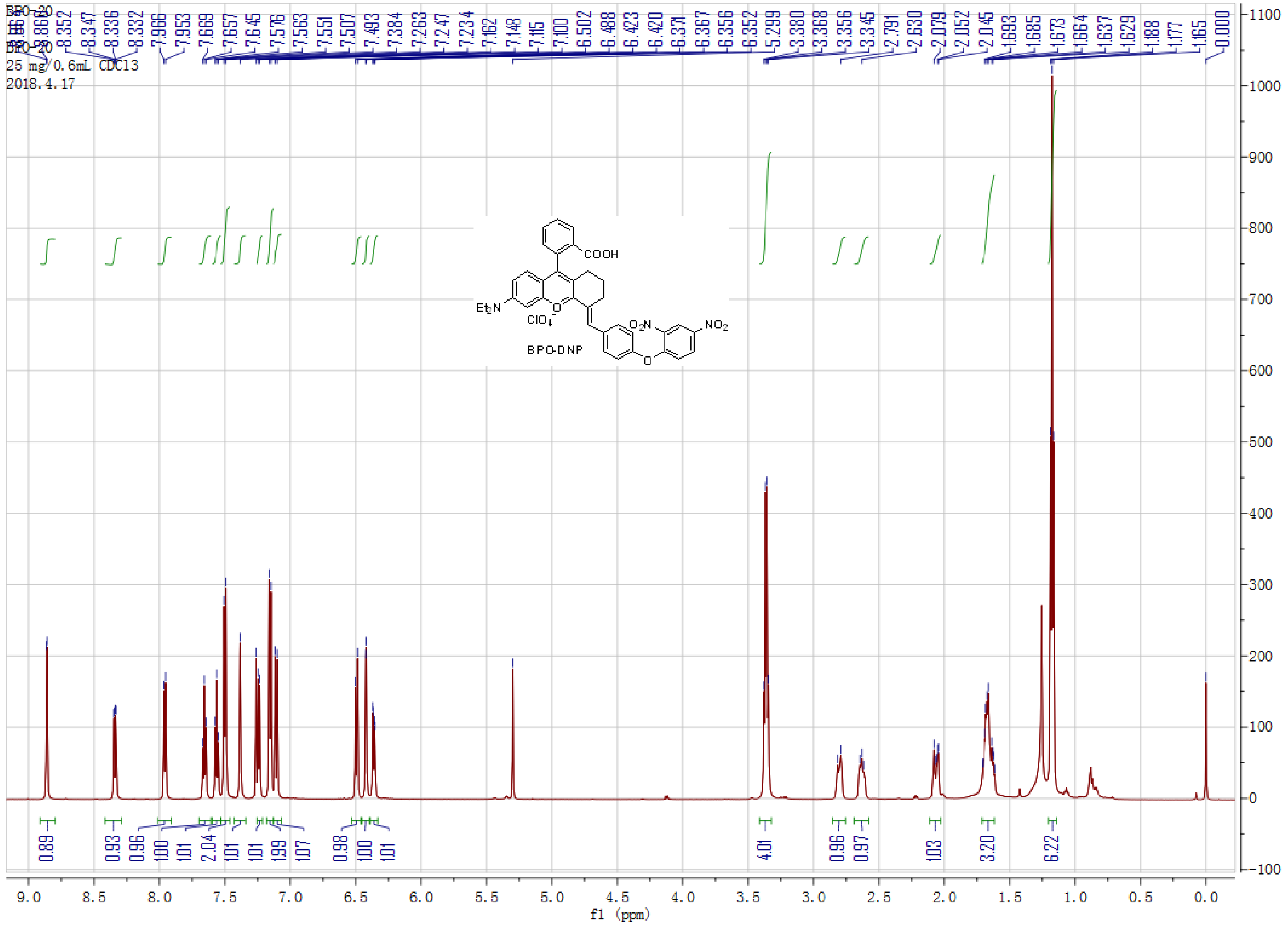

BPO-20
25 mg/0.6mL CDCl3
2018.4.17
8.865 8.352 8.347 8.336 8.332 7.966 7.953 7.669 7.657 7.645 7.576 7.563 7.551 7.507 7.493 7.384 7.263 7.247 7.234 7.162 7.148 7.115 7.100 6.502 6.488 6.423 6.420 6.371 6.367 6.356 6.352 5.299 3.380 3.368 3.356 3.345 2.791 2.630 2.079 2.052 2.045 1.693 1.685 1.673 1.664 1.637 1.629 1.188 1.177 1.165 0.000
COOH
Et2N
ClO4
O2N
NO2
BPO-DNP
0.89 0.93 0.96 1.00 1.01 2.04 1.01 1.01 1.99 1.07 0.98 1.00 1.01 4.01 0.96 0.97 1.03 3.20 6.22
f1 (ppm)

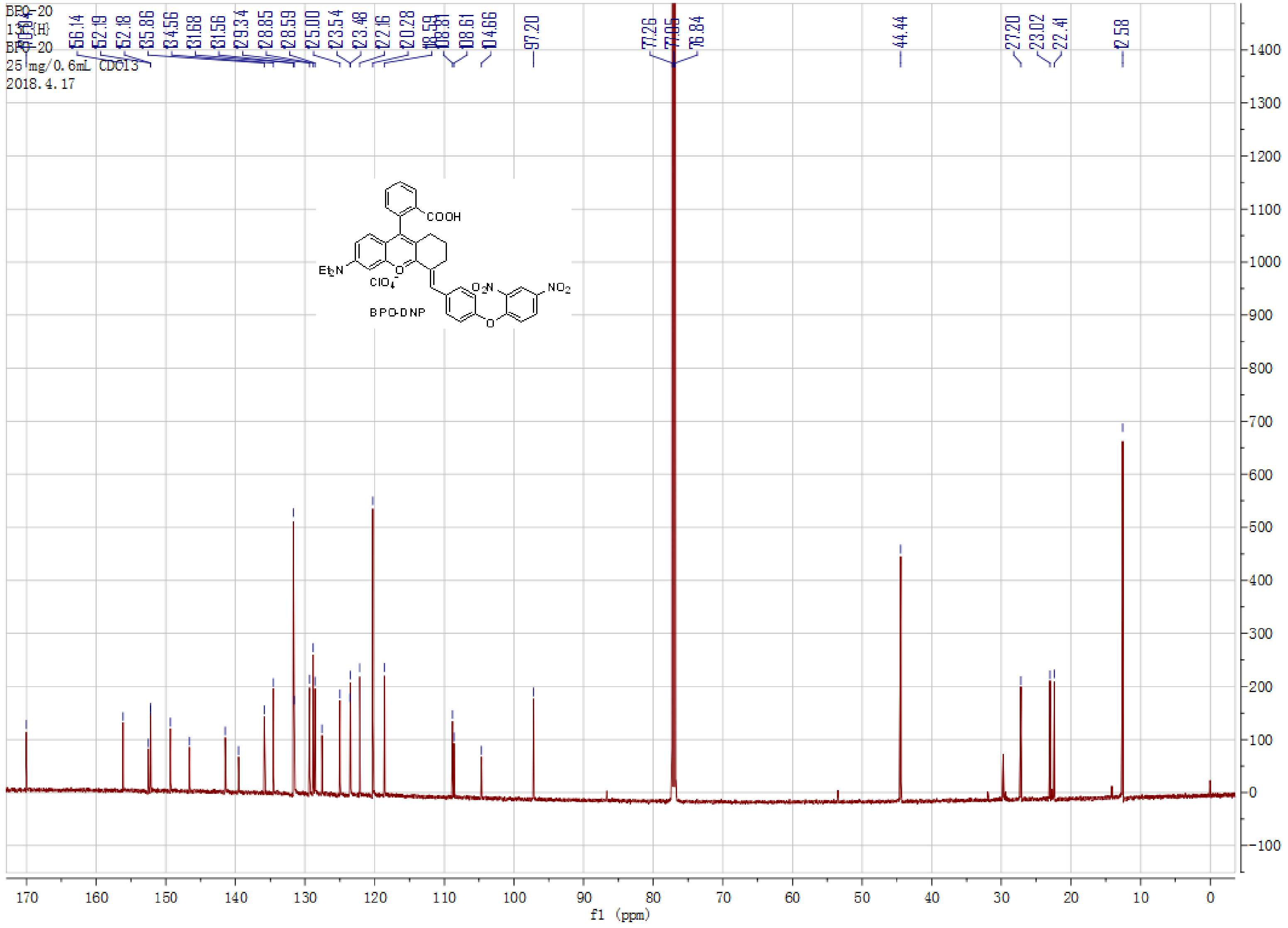
BPO-20
13C{H}
BPO-20
25 mg/0.6mL CDCl3
2018.4.17
156.14
152.19
152.18
135.86
134.56
131.68
131.56
129.34
128.85
128.59
125.00
123.54
123.48
122.16
120.28
108.61
104.66
97.20
77.26
76.84
44.44
27.20
23.02
22.41
12.58
COOH
Et2N
ClO4
O
O2N
NO2
BPO-DNP
f1 (ppm)

BPO-DNP

MS(E+)

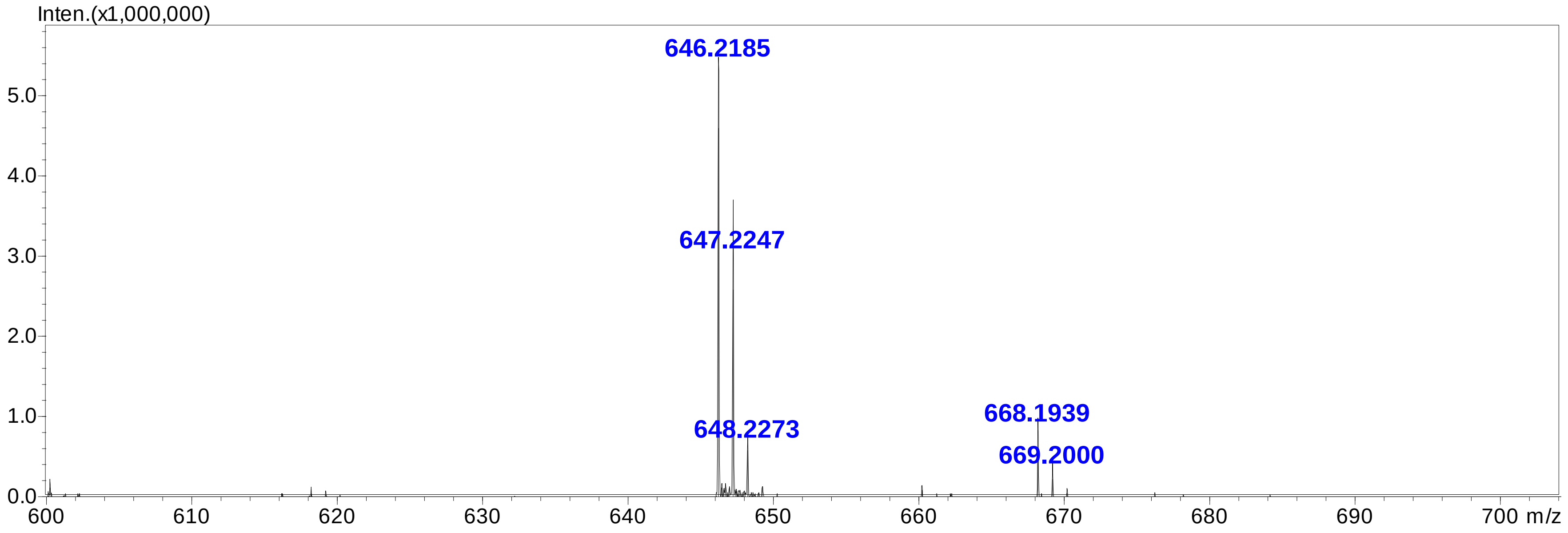
Inten.(x1,000,000)
5.0
4.0
3.0
2.0
1.0
0.0
646.2185
647.2247
648.2273
668.1939
669.2000
600
610
620
630
640
650
660
670
680
690
700 m/z
